# Nonthyroidal illness syndrome (NTIS) in severe COVID-19 patients: role of T3 on the Na/K pump gene expression and on hydroelectrolytic equilibrium

**DOI:** 10.1186/s12967-021-03163-z

**Published:** 2021-12-03

**Authors:** Salvatore Sciacchitano, Carlo Capalbo, Christian Napoli, Andrea Negro, Luciano De Biase, Adriano Marcolongo, Paolo Anibaldi, Valentina Salvati, Lea Petrella, Luca Merlo, Daniela Alampi, Elisa Alessandri, Chiara Loffredo, Alessandra Ulivieri, Luca Lavra, Fiorenza Magi, Alessandra Morgante, Leila B. Salehi, Claudia De Vitis, Rita Mancini, Flaminia Coluzzi, Monica Rocco

**Affiliations:** 1 Department of Clinical and Molecular Medicine, Sapienza University, Viale Regina Elena n. 324, 00161 Rome, Italy; 2 Laboratory of Biomedical Research, Niccolò Cusano University Foundation, Rome, Italy; 3 Department of Medical Oncology, Sant’Andrea University Hospital, 00189 Rome, Italy; 4 Department of Molecular Medicine, Sapienza University, Rome, Italy; 5 Department of Clinical and Surgical Translational Medicine, Sapienza University, Rome, Italy; 6 Heart Failure Unit, Department of Clinical and Molecular Medicine, Sapienza University of Rome, Rome, Italy; 7 General Director, Sant’Andrea Hospital, Rome, Italy; 8 Health Management Director, Sant’Andrea Hospital, Rome, Italy; 9 Scientific Direction, IRCCS Regina Elena National Cancer Institute, Rome, Italy; 10 Department of Methods and Models for Economics, Territory and Finance (MEMOTEF), Sapienza University of Rome, Rome, Italy; 11 Department of Statistical Sciences, Sapienza University of Rome, Rome, Italy; 12 Unit of Anesthesia, Intensive Care and Pain Medicine, Sant’Andrea University Hospital, Rome, Italy; 13 U.O.C. of Medical Genetics, Policlinic of Tor Vergata, Rome, Italy; 14 Department of Medico-Surgical Sciences and Biotechnologies, Sapienza University of Rome, Rome, Italy

**Keywords:** COVID-19, Nonthyroidal Illness Syndrome, NTIS, Bioelectrical Impedance Analysis, BIA, Hydration, Sodium/potassium exchangeable ratio, Na^+^/K^+^ pump gene

## Abstract

**Background:**

Nonthyroidal Illness Syndrome (NTIS) can be detected in many critical illnesses. Recently, we demonstrated that this condition is frequently observed in COVID-19 patients too and it is correlated with the severity the disease. However, the exact mechanism through which thyroid hormones influence the course of COVID-19, as well as that of many other critical illnesses, is not clear yet and treatment with T4, T3 or a combination of both is still controversial. Aim of this study was to analyze body composition in COVID-19 patients in search of possible correlation with the thyroid function.

**Methods and findings:**

We report here our experience performed in 74 critically ill COVID-19 patients hospitalized in the intensive care unit (ICU) of our University Hospital in Rome. In these patients, we evaluated the thyroid hormone function and body composition by Bioelectrical Impedance Analysis (BIA) during the acute phase of the disease at admission in the ICU. To examine the effects of thyroid function on BIA parameters we analyzed also 96 outpatients, affected by thyroid diseases in different functional conditions. We demonstrated that COVID-19 patients with low FT3 serum values exhibited increased values of the Total Body Water/Free Fat Mass (TBW/FFM) ratio. Patients with the lowest FT3 serum values had also the highest level of TBW/FFM ratio. This ratio is an indicator of the fraction of FFM as water and represents one of the best-known body-composition constants in mammals. We found an inverse correlation between FT3 serum values and this constant. Reduced FT3 serum values in COVID-19 patients were correlated with the increase in the total body water (TBW), the extracellular water (ECW) and the sodium/potassium exchangeable ratio (Na_*e*_:K_*e*_), and with the reduction of the intracellular water (ICW). No specific correlation was observed in thyroid patients at different functional conditions between any BIA parameters and FT3 serum values, except for the patient with myxedema, that showed a picture similar to that seen in COVID-19 patients with NTIS. Since the Na^+^/K^+^ pump is a well-known T3 target, we measured the mRNA expression levels of the two genes coding for the two major isoforms of this pump. We demonstrated that COVID-19 patients with NTIS had lower levels of mRNA of both genes in the peripheral blood mononuclear cells (PBMC)s obtained from our patients during the acute phase of the disease. In addition, we retrieved data from transcriptome analysis, performed on human-induced pluripotent stem cell-derived cardiomyocytes (hiPSC-CM)s treated with T3 and we demonstrated that in these cells T3 is able to stimulate the expression of these two genes in a dose-dependent manner.

**Conclusions:**

In conclusion, we demonstrated that measurement of BIA parameters is a useful method to analyze water and salt retention in COVID-19 patients hospitalized in ICU and, in particular, in those that develop NTIS. Our results indicate that NTIS has peculiar similarities with myxedema seen in severe hypothyroid patients, albeit it occurs more rapidly. The Na^+^/K^+^ pump is a possible target of T3 action, involved in the pathogenesis of the anasarcatic condition observed in our COVID-19 patients with NTIS. Finally, measurement of BIA parameters may represent good endpoints to evaluate the benefit of future clinical interventional trials, based on the administration of T3 in patients with NTIS.

## Introduction

In 1951, Douglas B. Brewer, by examining a man died after 10-year history of myxedema, reported that his tongue and his pericardium were infiltrated with a mixture of mucoproteins [1]. These proteins were unable to attract liquid because they had a too large molecular weight to exert osmotic effect. However, by acting as a hydrophilic gel, they were capable to attract salt and, consequently, water. More than 50 years ago, using a dilution method based on the administration of radioactive isotopes, the total body water and Na_*e*_ were found to be increased in myxedematous hypothyroid patients [2].

COVID-19 is responsible for the induction of a wide spectrum of clinical presentations, including fever, dry cough, asthenia, headache, myalgia, anosmia and diarrhea. The most severe forms of the disease are characterized by dyspnea and/or hypoxemia that can quickly progress to septic shock, uncorrectable metabolic acidosis and coagulation disorders. In these patients, hospital admission is needed for intensive care, and death may occur in 4.3–15% of them [3]. COVID-19 mainly affects the lung with injuries responsible for the occurrence of acute respiratory distress syndrome (ARDS) [4]. During COVID-19, edema may be present in many different tissues, including myocardial and pulmonary tissues and may be associated with pleural [5] as well as pericardium effusions [6]. In addition, in some patients, the occurrence of encephalitis with brain edema has been reported [7]. Finally, COVID-19 has been associated also with acral edema [8]. There have been some attempts to identify, during the acute phase of the disease, specific parameters of body composition using the bioelectric impedance analysis (BIA), mostly focused on nutrition, possibly associated with the disease severity. They included measurements of fat mass, visceral fat area, and fat-free mass. However, a recent observational cross-sectional cohort study failed to detect significant association between body composition and disease severity [9]. In another retrospective study, although the clinical utility of BIA was recognized in predicting the length-of-staying (LOS), bioimpedance did not seem to add further predictive value [10].

We recently reported that COVID-19 patients frequently presented the reduction of the free triiodothyronine (FT3) serum levels [11]. This condition is named Low T3 Syndrome or Nonthyroidal Illness Syndrome (NTIS). We identified specific immunological and genetic signatures of this syndrome in COVID-19 patients and we reported that this condition represented a new marker of disease severity. Our results were in agreement with those presented by others [12]. In addition, this syndrome was found to be associated with increased lethality risk in COVID-19 patients [13]. The exact mechanism at the basis of this syndrome and its clinical significance are not well defined yet. In particular, it is not clear whether the changes in thyroid hormone levels in NTIS are associated with maladaptation rather than beneficial adaptation [14]. As a consequence, there is controversy about treatment indication of this condition, using either T3, T4 or a combination of both [15].

In the present study, we evaluated the impact of NTIS on the nutritional and hydration conditions of COVID-19 patients hospitalized in the ICU of our University Hospital in Rome, by analyzing body composition by means of the BIA. Our results indicate that the occurrence of low FT3 serum levels is associated with altered distribution of salt and water in the body as a consequence of reduced thyroid hormone activity at the periphery, and suggest that BIA could represent an easy and rapid method to obtain optimal endpoints for future clinical trials, focused to the evaluation of the potential benefit of the treatment with T3 in patients with NTIS.

## Materials and methods

### Subjects recruitment

The study has been conducted at the Sant’Andrea University Hospital, designated as one of the referral Hospitals in Rome, Italy for COVID-19 and that adopted a dedicated integrated care pathway to manage COVID-19 patients [16]. During the last wave of SARS-CoV-2 outbreak, we examined a total of 74 critically ill patients, hospitalized at the ICU, that were found to be positive for the presence of the SARS-CoV-2 virus in the nasal and oropharyngeal swabs, according to the WHO recommendations [17]. In particular, two real-time reverse-transcription polymerase chain reaction (rRT-PCR) detection kits have been used, namely the Allplex™ 2019-nCoV Assay (Seegene, Seul, Republic of Korea) and the RNA Detection kit (DAAN Gene Co. LTD, Guangzhou, Guandong, China), both CE-approved. The diagnosis has been considered positive in the presence of at least two of the three genes considered (E, RdRP and N genes) for the kit by Seegene and of the two genes considered (ORF1ab and N genes) for the kit by DAAN. In 42 patients COVID-19 was associated with NTIS, while in 32 of them, used as internal control group, there was no evidence of NTIS. A group of 96 non-COVID-19 outpatients, with no critical illnesses and affected by several different thyroid disease was also used as external control to verify the specificity of the observed effects in different thyroid functional states. In particular, we analyzed 19 patients with subclinical hyperthyroidism, characterized by TSH serum levels < 0.35 µIU/ml, 57 patients with normal thyroid function, with TSH serum levels included between 0.35 and 4.0 µIU/ml, 12 patients with subclinical hypothyroidism, with TSH levels ≥ 4 µIU/ml and 7 patients with overt hypothyroidism, showing TSH levels ≥ 10 µIU/ml. In addition, we examined 1 patient with severe hypothyroidism and myxedema, presenting TSH serum level > 100 µIU/ml.

### Thyroid hormone function tests

The FT3, FT4 and TSH serum levels were assayed using a Chemiluminescent Microparticle Immunoassay (CMIA), an immunoassay analyzer (ARCHITECT i1000SR, Abbott Lab., Abbott Park, IL, USA) and specific, dedicated diagnostic kits (ARCHITECT Free T3, FT4 and TSH assay, Abbott Lab., Abbott Park, IL, USA). Conventional reference intervals for FT3, FT4 and TSH were 1.71–3.71 pg/ml, 0.7–1.48 ng/dl and 0.35–4.0 µIU/ml, respectively.

### Electrolyte measurements

The measurement of Na^+^ and K^+^ in the serum was performed using the Architect ICT kit and the Abbott Architect C16000 Clinical Chemistry Analyzer (Abbott Lab. Abbott Park, IL, USA) according to the manufacturer’s instructions. Conventional reference intervals for Na^+^ and K^+^ were 136 to 145 mmol/L and 3.5 to 5.1 mmol/l respectively.

### Analysis of body composition by bioelectrical impedance analysis (BIA)

We analyzed body composition of our patients using a single-frequency Bioelectrical Impedance Analysis (SF-BIA), namely the BIA 101 analyzer (Akern Srl, Pontassieve, Firenze, Italy). The measurements were taken while the subjects were supine in bed. Patients were exposed to the passage of a painless low amplitude, alternating electrical current of 50 kHz, applied through cables connected to electrodes (Biatrodes electrodes, Jatreia, Pescantina, Verona, Italy), placed in contact with the skin of the right hand and of the right foot. The BIA instrument was used according to the manufacturer’s instruction and always by the same operator. Statures were measured to the nearest 0.5 cm and weights were measured to the nearest 0.1 kg using bed weighing scales. We measured three parameters, namely the resistance (Rz), the reactance (Xc) and the phase angle (PhA). Impedance (Z) is represented by a complex number, i.e., a combination of resistance Rz, the opposition to the flow of the injected current at any current frequency trough intra and extra-cellular ionic solutions, and reactance Xc, the capacitive component of cell membranes and tissue interfaces. The arc tangent of the proportion between the two components is called phase angle (PhA). Based on these parameters, we obtained estimates of a number of BIA parameters, including the body cell mass (BCM), the total body water (TBW), the extra cellular water (ECW), the intra cellular water (ICW), the Na:K exchange rate (Na_*e*_:K_*e*_), the fat-free mass (FFM) and the fat mass (FM). In addition, we calculated the hydration and nutrition state, using the following formulas: hydration = TBW/FFM and nutrition = mg/24 h/htm [The BIA compendium, 3 (http://www.data-input.de)]. BIA analysis was performed using the Bodygram PRO software (vers. 3.0, Jatreia, Pescantina, Verona, Italy).

### SOFA score

The Sequential Organ Failure Assessment (SOFA) score, a mortality prediction score based on the degree of dysfunction of six organ systems, has been calculated using the following variables: PaO_2_/FiO_2_ measured in mmHg, Platelets counted in × 10^3^/μl, the Glasgow Coma Scale from grade 0 to + 4, the bilirubin, measured in mg/dl, the mean arterial pressure in mmHg and the creatinine in mg/dl. The SOFA score has been recently used to evaluate the severity of the clinical conditions in COVID-19 patients [18] and in those with NTIS too [11].

### Lethality

We registered and reported lethality of COVID-19 patients, hospitalized in ICU during the study period and we correlated it with the FT3 serum levels measured at admission.

### Ethics and statistical analysis

The study was approved by the Institutional Ethical Committee of our University La Sapienza of Rome, Italy (RIF. CE 5773_2020, Prot. # 52SA_2020, and its subsequent substantial amendment RIF. CE 5773_2020, Prot. # 171SA_2020), on the basis that it complied with the declaration of Helsinki and that the protocol followed existing good clinical practice guidelines and informed consent was obtained from each individual.

We analyzed data from COVID-19 patients (n = 74) and patients that were not infected, affected by thyroid diseases at different functional states (n = 96). The considered clinical variables include gender, age, BMI, FT3, FT4, TSH, ECW, PhA, Na_*e*_:K_*e*_ ratio and hydration.

For continuous variables, differences between means in the two groups were evaluated using the two-sample t-test while the nonparametric Wilcoxon test was used for non-normally distributed variables. Categorical variables were expressed as absolute frequencies while continuous variables were given as mean ± standard deviation (St. Dev.). In order to identify potential factors associated with FT3 levels, we estimated a multivariable linear regression model on all patients in the sample where we add the COVID-19 dummy variable among the covariates.

To avoid violations of the assumptions underlying the model, we log-transformed FT4 and TSH. Finally, both univariable and multivariable logistic regression models were estimated on COVID-19 Positive patients to evaluate potential factors associated with low levels of FT3 (FT3 ≤ 1.7 pg/ml). In the multivariable analysis, in order to select the predictors to be included in the final model, we employed a stepwise selection procedure by iteratively adding and removing candidate predictors in the regression model based on the Akaike Information Criteria (AIC). Statistical significance of the regression coefficient was assumed if the *p*-value was less than 0.05. The statistical analysis has been performed using the R software (R Development Core Team, 2009), version 4.0.2.

### Isolation of PBMC from COVID-19 patients and RNA extraction, cDNA synthesis and RT-qPCR

We were able to collect and isolate PBMCs from 47 COVID-19 patients (28 with and 19 without NTIS). In these patients, PBMC samples, BIA and measurement of FT3 serum levels were performed in the same day. Collection and isolation of PBMCs were performed as previously described [11]. Total RNA from PBMC samples was extracted using the Qiazol Lysis Reagent (Qiazol) and miRNeasy Mini Kit (Qiazol) following the manufacturer’s instructions [19]. The first-strand cDNA was synthesized according to the instructions for the kit (Takara). Real time quantitative PCR (RT-qPCR) was performed using TaqMan Fast Advanced Master mix (Applied Biosystems) on an ABI Prism 7900 apparatus (Applied Biosystems). We analyzed the mRNA expression levels of the two genes that encode the two major isoforms of the Na^+^/K^+^ pump, namely the ATPase Na^+^/K^+^ transporting subunit α 1 (ATP1A1) and the ATPase Na^+^/K^+^ transporting subunit β 1 (ATP1B1). The following TaqMan Gene Expression Assay (FAM) (Thermo Fisher Scientific) were used: ATP1A1 (Hs00933601_m1); ATP1B1 (Hs00426868_g1). The mRNA expression was normalized using the ∆∆Ct method for relative quantification. The expression of β-actin was used as internal control, as specified. Relative mRNA expression was calculated using the comparative Ct method (10-deltaCT) [20].

### ATP1A1 and ATP1B1 expression in hiPSC-derived cardiomyocytes (hiPSC-CM)

Expression data regarding ATP1A1 and ATP1B1 genes among untreated and treated with T3 at 1 nM and 100 nM were retrieved at NCBI GEO database, relating to RNAseq experiments performed to investigate the T3 effects on hiPSC-CMs obtained from circulating blood fibroblasts of a Caucasian 18 years old female and reprogrammed by retroviral transduction (accession GSE172348, GEO reviewer token: sngtkywkbjwxrip).

## Results

We analyzed a total of 74 patients admitted to the ICU of our University Hospital in Rome during the last wave of COVID-19 pandemic, from the beginning of March to the end of May 2021. We examined also 96 outpatients affected by thyroid disease, at different thyroid functional conditions. These patients were included in the study as external controls to assess the effects of thyroid hormonal status on BIA parameters in patient without critical illnesses. The epidemiological and clinical data of COVID-19 patients are reported (Table 2). In addition, we report also the epidemiological and clinical data of the non-COVID-19 outpatients with thyroid diseases (Table 1). Among the COVID-19 patients, the occurrence of NTIS was observed in 42 of them (56.7%). COVID-19 patients with low FT3 values were further subdivided into those with values of FT3 ranging from 1.1 to 1.7 pg/ml (n. 29) and those with very low FT3 serum values ≤ 1.1 pg/ml (n. 13). SOFA score at admission was slightly higher in the group of COVID-19 patients with low FT3 serum values (mean value = 4 ± 1.2) compared to the group of patients with normal FT3 serum values (mean value = 3 ± 1.6). Hypertension was the most frequently associated diseases in all groups of COVID-19 patients, followed by diabetes and obesity, with no significant differences related to their FT3 serum values (Table 2). As previously demonstrated, SOFA score correlates with the FT3 serum levels in our COVID-19 patients [11]. We observed, in fact, a score ≥ 4 in 17 out of 42 patients (40.5%) in the COVID-19 group of patients with low FT3 serum values as compared to 9 out of 32 patients (28%) with normal FT3 serum values. FT3 serum levels were also correlated to lethality. During the study period, 26 patients died during hospitalization for an overall mortality rate of 35%. The lethality rate was higher among the group of COVID-19 patients with low FT3 serum values at admission (47.6%), compared to that registered among COVID-19 patients that showed normal FT3 serum values at admission (18.8%).

**Table 1:** Non-COVID-19 outpatients with thyroid diseases

	Hyper Sub (n. 19) Mean (St. Dev.)	Euthyroid (n. 57) Mean (St. Dev.)	Hypo Sub (n. 12) Mean (St. Dev.)	Hypo Overt (n. 7) Mean (St. Dev.)	Myxedema (n. 1)	Total (n. 96) Mean (St. Dev.)
Sex (F/M)	17/2	50/7	12/0	6/1	0/1	85/11
Age (years)	55.7 (± 14.3)	54.8 (± 14.8)	49.2 (± 17.7)	53.4 (± 20.3)	78	54.4 (± 15.5)
BMI	25.8 (± 5.1)	26.9 (± 5.7)	24.4 (± 6.2)	27.3 (± 9.0)	27.6	26.4 (± 5.9)
FT3 (pg/ml)	3.2 (± 0.8)	2.9 (± 0.4)	2.7 (± 0.5)	2.0 (± 0.6)	1.7	2.9 (± 0.6)
FT4 (ng/dl)	1.4 (± 0.3)	1.2 (± 0.3)	1.1 (± 0.3)	1.3 (± 1.1)	0.4	1.2 (± 0.4)
TSH (μIU/ml)	0.1 (± 0.1)	1.7 (± 0.8)	6.3 (± 1.7)	22.1 (± 10.1)	100.0	4.5 (± 11.6)

**Table 2:** COVID-19 patients

	Normal FT3 (n. 32) Mean (St. Dev.)	Low FT3 (n. 29) Mean (St. Dev.)	Very low FT3 (n. 13) Mean (St. Dev.)	Total (n. 74) Mean (St. Dev.)
Sex (F/M)	17/15	14/15	7/6	38/36
Age (years), mean (st. dev.)	63.5 (± 13.8)	65.4 (± 13.7)	65.1 (± 10.1)	64.5 (± 13.1)
BMI, mean (st. dev.)	29.5 (± 7.5)	27.1 (± 4.2)	31.4 (± 7.6)	28.9 (± 6.6)
FT3 (pg/ml), mean (st. dev.)	2.2 (± 0.4)	1.5 (± 0.1)	1.0 (± 0.0)	1.7 (± 0.6)
FT4 (ng/dl), mean (st. dev.)	1.0 (± 0.2)	1.0 (± 0.2)	0.9 (± 0.2)	1.0 (± 0.2)
TSH (μIU/ml), mean (st. dev.)	1.4 (± 1.3)	0.8 (± 0.9)	0.6 (± 0.8)	1.0 (± 1.1)
Comorbidity (at least one) n. (%)	26 (81.3)	24 (82.7)	13 (100)	63 (85.1)
Hypertension, n. (%)	23 (71.9)	17 (58.6)	11 (84.6)	51 (68.9)
Diabetes, n. (%)	15 (46.9)	8 (27.6)	6 (46.2)	29 (39.2)
Obesity, n. (%)	9 (28.1)	4 (13.8)	5 (38.5)	18 (24.3)
Malignancy, n. (%)	2 (6.3)	6 (20.7)	8 (19)	16 (21.6)
Ischemic heart disease, n. (%)	8 (25.0)	5 (17.2)	1 (7.7)	14 (18.9)
Arrhythmogenic cardiac diseases, n. (%)	1 (3.1)	2 (6.9)	1 (7.7)	4 (5.4)
Autoimmune disease, n. (%)	2 (6.3)	2 (6.9)	0 (0)	4 (5.4)
Chronic obstructive pulmonary disease, n. (%)	2 (6.3)	1 (3.4)	0 (0)	3 (4.0)
Psychiatric disease, n. (%)	1 (3.1)	1 (3.4)	1 (7.7)	3 (4.0)

### Lower FT3 serum values in COVID-19 patients are associated with marked salt and water retention

**Fig. 1:**
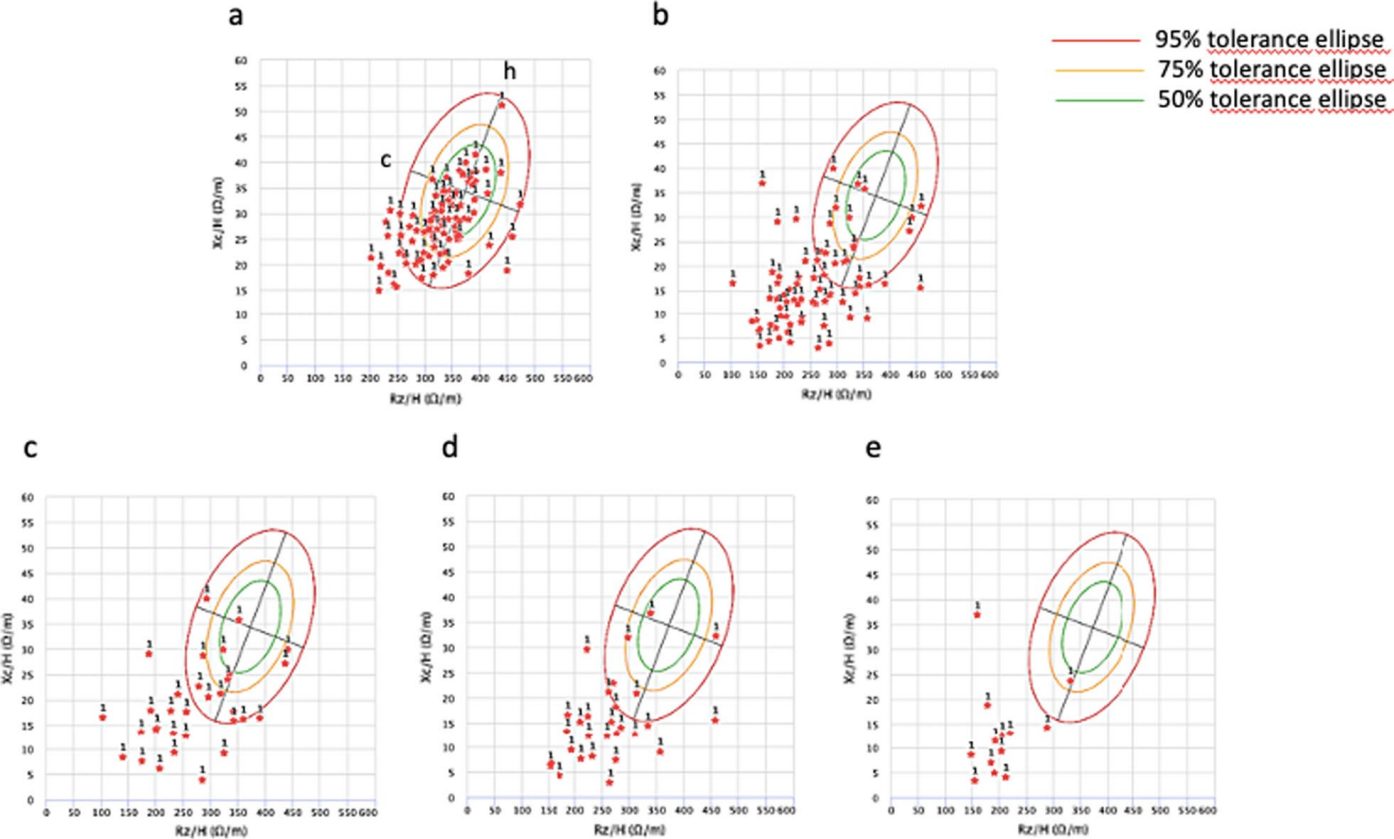
*R*-*Xc* (resistance versus reactance) graphs of bioelectrical impedance vector analysis data of patients with COVID-19 compared to non-COVID-19 thyroid patients. Red stars indicate individual measurement at admission. The 95%, 75% and 50% tolerance ellipses are shown. The lower axis (c) indicates cellularity (above, to the left, indicate more body cell mass and below, to the right, less body cell mass), while the largest axis (h) indicates hydration (dehydration towards the upper pole, hyperhydration with apparent edema toward the lower pole). **a** Non COVID-19 control patients (n. 95); **b** COVID-19 patients (n. 74); **c** COVID-19 patients with FT3 serum values > 1.7 pg/ml (n. 32); **d** COVID-19 patients with FT3 serum values ranging from 1.1 to 1.7 pg/ml (n. 29); **e** COVID-19 patients with FT3 serum values < 1.1 pg/ml (n. 13)

The *R*-*Xc* (resistance versus reactance) graphs of vector BIA are reported in Fig. 1. We observed that our COVID-19 patients showed, displacement of vectors toward the downward direction of the largest axis (h) of the ellipse, indicating high prevalence of anasarcatic status in these patients (Fig. 1b), compared to our control patients that were almost all included inside the 95% tolerance ellipse (Fig. 1a). When we analyzed the results obtained in our COVID-19 patients with respect to the FT3 serum values we found that COVID-19 patients with low (> 1.1. and ≤ 1.7 pg/ml) or very low FT3 serum levels (≤ 1.1 pg/ml) presented a progressive shift toward the lower pole of largest axis h, indicating the occurrence of anasarcatic conditions in almost all of them (Fig. 1d, e). These results indicated that the anasarcatic condition was associated with the reduction in the FT3 serum levels.

**Fig. 2:**
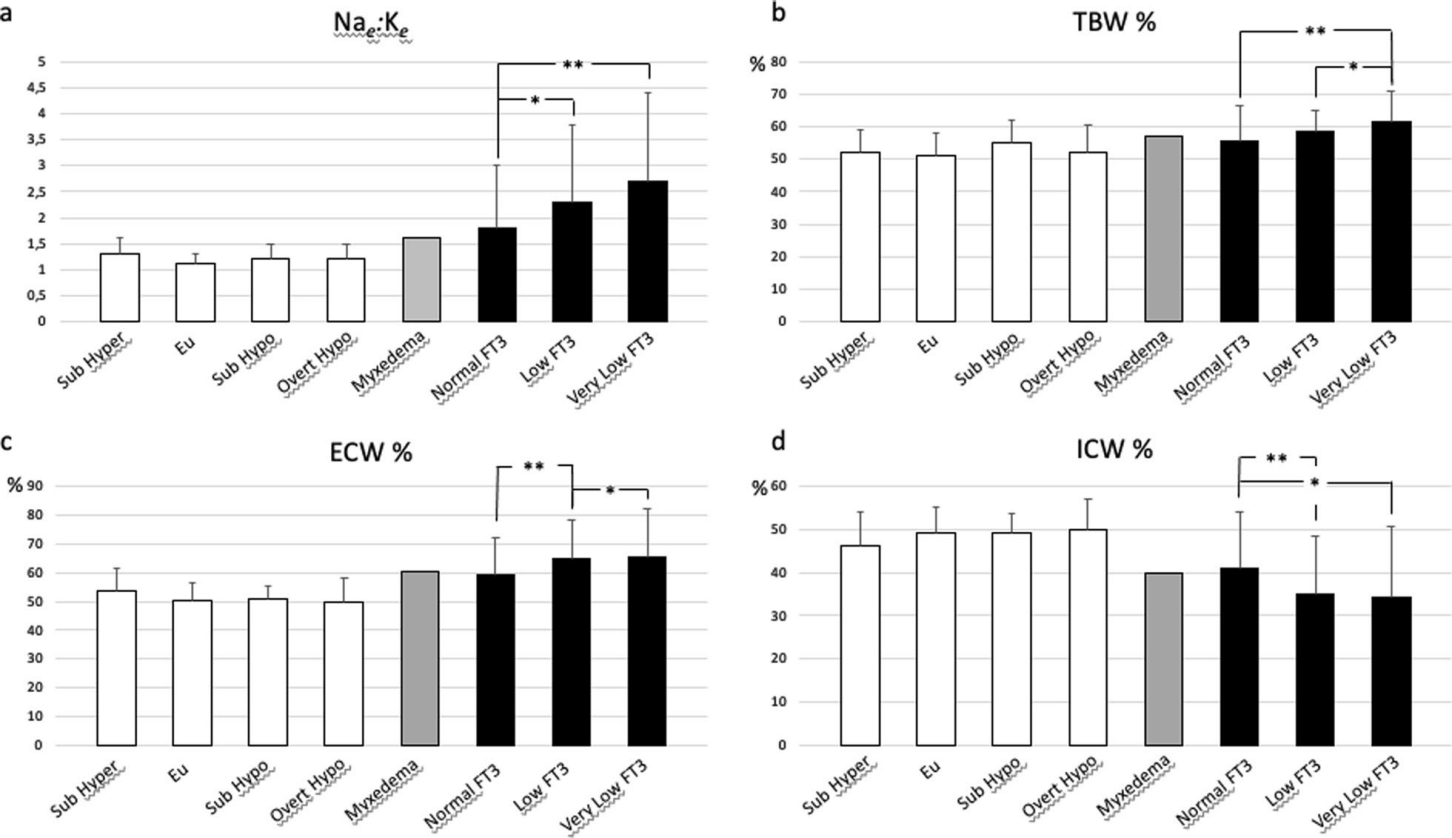
Analysis of BIA parameters in COVID-19 (black bars) and in non-COVID-19 outpatients with thyroid diseases (white bars). The non-COVID-19 outpatient with severe hypothyroidism and myxedema is also displayed (grey bar). Na_*e*_:K_*e*_ ratio, sodium/potassium exchangeable ratio; TBW %, percentage of total body water; ECW %, percentage of extracellular water; ICW %, percentage of intracellular water. *p < 0.05

We then checked whether there could be any correlation between the FT3 serum levels and any of the parameters measured by BIA. The mean values of BIA analysis in our COVID-19 patients as well as the non-COVID-19 outpatients are reported in Tables 3, 4. In COVID-19 patients, low and very low FT3 serum values were correlated with reduced values of PhA, increased amount of TBW and ECW and reduced content of ICW (Table 6). FT3 serum values were also correlated with the Na_*e*_:K_*e*_ ratio (Table 6) (Fig. 2a).

**Table 3:** BIA measurements and thyroid function in non-COVID-19 outpatients with thyroid diseases

	Hyper Sub (n. 19) Mean (St Dev.)	Euthyroid (n. 57) Mean (St Dev.)	Hypo Sub (n. 12) Mean (St. Dev.)	Hypo Overt (n. 7) Mean (St. Dev.)	Myxedema (n. 1)	Total (n.96) Mean (St. Dev.)
FT3 (pg/ml)	3.2 (± 0.8)	2.9 (± 0.4)	2.7 (± 0.5)	2.0 (± 0.6)	1.66	2.9 (± 0.6)
FT4 (ng/dl)	1.4 (± 0.3)	1.2 (± 0.3)	1.1 (± 0.3)	1.3 (± 1.1)	0.37	1.2 (± 0.4)
TSH (μIU/ml)	0.1 (± 0.1)	1.7 (± 0.8)	6.3 (± 1.7)	22.1 (± 10.1)	100	4.5 (± 11.6)
Rz	561.5 (± 96)	540.3 (± 78.3)	552.3 (± 104.6)	522.6 (± 87.8)	459	543.9 (± 85.4)
Xc	44.7 (± 9.8)	49.0 (± 17.1)	49.6 (± 16.0)	47.7 (± 10.4)	29	47.9 (± 15.3)
PhA	4.7 (± 1.1)	5.1 (± 1.2)	5.1 (± 0.8)	5.3 (± 1.3)	3.6	5.0 (± 1.2)
FFM	45.2 (± 8.5)	48.6 (± 7.4)	43.8 (± 3.8)	51.8 (± 9.5)	48.7	47.5 (± 7.5)
TBW	33.9 (± 6.4)	36.1 (± 6.1)	32.6 (± 3.1)	38.5 (± 6.4)	38.9	35.4 (± 6.0)
ECW	18.0 (± 2.9)	18.4 (± 3.9)	16.7 (± 2.6)	18.9 (± 1.3)	23.4	18.2 (± 3.5)
BCM	20.9 (± 7.0)	23.6 (± 5.5)	21.1 (± 2.7)	26.1 (± 8.3)	18.7	22.9 (± 5.9)
FM	20.3 (± 8.2)	23.2 (± 11.8)	16.5 (± 7.7)	24.5 (± 16.6)	19.3	21.9 (± 11.1)
Na_*e*_:K_e_ ratio	1.3 (± 0.3)	1.1 (± 0.2)	1.2 (± 0.3)	1.2 (± 0.3)	1.57	1.2 (± 0.3)
FM%	30.4 (± 8.9)	30.9 (± 9.4)	26.3 (± 8.2)	30.0 (± 10.2)	28.4	30.1 (± 9.1)
FFM%	69.6 (± 8.9	69.1 (± 9.4)	73.8 (± 8.2)	70.0 (± 10.2)	71.6	69.9 (± 9.1)
TBW%	52.2 (± 6.7)	51.2 (± 6.7)	55.0 (± 7.0)	52.2 (± 8.3)	57.2	52.0 (± 6.9)
ECW%	53.8 (± 7.7)	50.9 (± 6.1)	50.9 (± 4.7)	49.9 (± 7.0)	60.2	51.5 (± 6.4)
ICW%	46.2 (± 7.7)	49.1 (± 6.1)	49.1 (± 4.7)	50.1 (± 7.0)	39.8	48.5 (± 6.4)
MM	26.5 (8.0)	29.6 (± 6.2)	26.5 (± 3.0)	32.5 (± 9.5)	24.8	28.7 (± 6.7)
MM%	40.3 (8.5)	42.2 (± 8.8)	44.6 (± 5.3)	43.0 (± 7.8)	36.5	42.1 (± 8.3)
BCMI	8.1 (± 2.4)	8.8 (± 1.7)	8.5 (± 1.3)	9.3 (± 3.1)	7.6	8.6 (± 1.9)
ECM	24.3 (± 3.2)	25.0 (± 4.3)	22.7 (± 3.0)	25.7 (± 1.9)	30	24.7 (± 3.9)
TBW/FFM (hydration)	75.1 (± 3.5)	74.3 (± 4.6)	74.5 (± 3.1)	74.5 (± 2.4)	79.9	74.5 (± 4.1)
Nutrition	628.7 (± 206.6)	695.1 (± 148.1)	639.2 (± 86.3)	754.0 (± 262.0)	605.1	678.3 (± 166.0)
SMI	7.9 (± 1.7)	8.2 (± 1.4)	8.1 (± 1.1)	8.5 (± 2.3)	10.1	8.2 (± 1.5)
SMM	26.5 (± 8.0)	29.6 (± 6.2)	26.5 (± 3.0)	32.5 (± 9.5)	24.8	28.7 (± 6.7)
ASMM	16.7 (± 4.2)	18.3 (± 3.7)	16.0 (± 1.5)	20.1 (± 5.1)	17.9	17.8 (± 3.8)
FMI	8.1 (± 3.8)	8.7 (± 4.5)	6.8 (± 3.8)	8.8 (± 6.3)	7.8	8.4 (± 8.6)
FFMI	17.6 (± 2.6)	18.1 (± 1.9)	17.6 (± 2.6)	18.5 (± 3.8)	19.8	18.0 (± 2.3)
SPA	− 1.4 (± 1.2)	− 1.0 (± 1.2)	− 1.3 (± 0.8)	− 0.8 (± 1.0)	− 1.89	− 1.1 (± 1.2)

**Table 4:** BIA measurements and thyroid function in COVID-19 patients

	Normal FT3 (n. 32) Mean (St. Dev.)	Low FT3 (n. 29) Mean (St. Dev.)	Very low FT3 (n. 13) Mean (St. Dev.)	Total (n. 74) Mean (St. Dev.)
FT3 (pg/ml)	2.2 (± 0.4)	1.5 (± 0.1)	1.0 (± 0.0)	1.7 (± 0.6)
FT4 (ng/dl)	1.0 (± 0.2)	1.0 (± 0.2)	0.9 (± 0.2)	1.0 (± 0.2)
TSH (μIU/ml)	1.4 (± 1.3)	0.8 (± 0.9)	0.6 (± 0.8)	1.0 (± 1.1)
Rz	453.6 (± 123.8)	445.0 (± 121.8)	339.5 (± 81.6)	430.2 (± 122.8)
Xc	31.6 (± 14.5)	26.0 (± 14.2)	20.8 (± 14.9)	27.5 (± 14.8)
PhA	4.1 (± 1.9)	3.3 (± 1.6)	3.6 (± 3.0)	3.7 (± 2.0)
FFM	55.4 (± 15.8)	53.1 (± 12.3)	59.3 (± 11.8)	55.2 (± 13.8)
TBW	46.0 (± 14.8)	45.4 (± 11.0)	52.3 (± 10.3)	46.9 (± 12.8)
ECW	26.6 (± 8.5)	29.4 (± 8.9)	34.4 (± 11.9)	29.1 (± 9.6)
BCM	23.0 (± 13.0)	18.1 (± 10.2)	19.7 (± 14.5)	20.5 (± 12.3)
FM	28.1 (± 17.4)	24.3 (± 8.2)	27.3 (± 16.3)	26.4 (± 14.2)
Na_*e*_:K_e_ ratio	1.8 (± 1.2)	2.3 (± 1.5)	2.7 (± 1.7)	2.1 (± 1.4)
FM%	32.6 (± 11.9)	31.4 (± 8.1)	30.1 (± 10.4)	31.7 (± 10.2)
FFM%	67.4 (± 11.9)	68.6 (± 8.1)	69.9 (± 10.4)	68.3 (± 10.2)
TBW%	55.7 (± 10.7)	58.5 (± 6.3)	61.7 (± 9.4)	57.8 (± 9.1)
ECW%	59.2 (± 13.1)	65.0 (± 13.4)	65.6 (± 16.3)	62.6 (± 14.0)
ICW%	40.9 (± 13.1)	35.0 (± 13.4)	34.4 (± 16.3)	37.4 (± 14.0)
MM	30.1 (± 14.9)	24.9 (± 11.3)	27.6 (± 15.4)	27.6 (± 13.7)
MM%	36.0 (± 15.4)	32.0 (± 13.1)	32.1 (± 16.4)	33.7 (± 14.6)
BCMI	8.0 (± 4.1)	6.3 (± 3.5)	7.2 (± 5.3)	7.2 (± 4.1)
ECM	32.4 (± 8.3)	35.0 (± 9.0)	39.5 (± 13.1)	34.7 (± 9.8)
TBW/FFM (hydration)	82.4 (± 7.5)	85.7 (± 7.6)	88.5 (± 7.0)	84.8 (± 7.7)
Nutrition	671.8 (± 374.7)	528.2 (± 295.4)	583.8 (± 419.3)	600.0 (± 355.4)
SMI	10.4 (± 3.7)	10.5 (± 2.9)	13.2 (± 2.9)	10.9 (± 3.4)
SMM	30.1 (± 14.9)	24.9 (± 11.3)	27.6 (± 15.4)	27.6 (± 13.7)
ASMM	23.4 (± 9.5)	22.4 (± 6.7)	26.7 (± 6.6)	23.6 (± 8.0)
FMI	10.1 (± 6.1)	8.5 (± 2.8)	9.9 (± 6.3)	9.4 (± 5.1)
FFMI	19.5 (± 4.0)	18.5 (± 3.4)	21.4 (± 3.7)	19.4 (± 3.8)
SPA	− 1.6 (± 2.2)	− 2.5 (± 1.4)	− 2.0 (± 2.8)	− 2.0 (± 2.0)

**Fig. 3:**
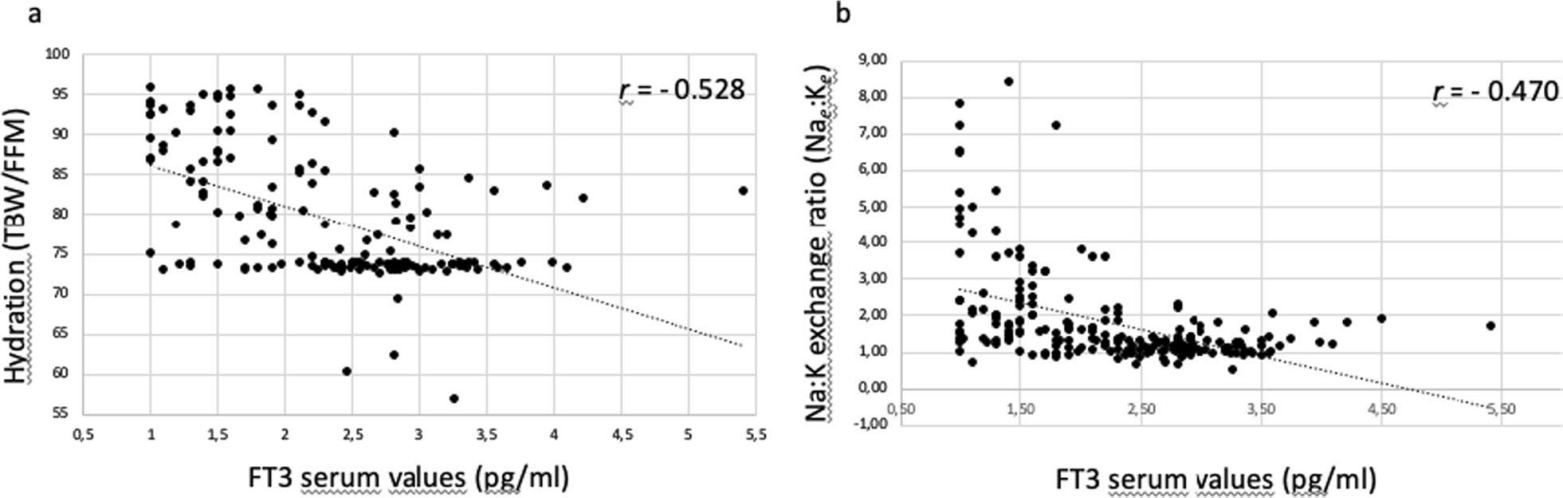
Correlation between FT3 serum levels and hydration (**a**) and between FT3 serum levels and Na_*e*_:K_*e*_ ratio (**b**). The coefficient of correlation *r* is reported. In our lab FT3 values under 1.0 pg/ml are not measured. They are classified as < 1

Hydration, measured as the ratio of total body water (TBW) to fat-free body mass (FFM), is an exceptionally stable constant. In mammals, it is approximately 0.73 and represents a cornerstone in the body-composition research field [21]. We found an inverse significant correlation between FT3 serum levels and the hydration status (Fig. 3a). In non-COVID-19 outpatients, the TBW/FFM ratio remained stable and didn’t changed with the different thyroid function conditions considered. The TBW/FFM ratio was slightly elevated only in the single patient affected by severe hypothyroidism and myxedema (Table 3). Conversely, the TBW/FFM ratio was clearly increased in COVID-19 patients and the increase was more evident in those with low and very low FT3 serum values (Table 4). We then analyzed whether water retention was associated with salt retention. The results of the vector BIA analysis of the sodium/potassium exchange ratio (Na_*e*_:K_*e*_), in both COVID-19 and non-COVID-19 patients, are reported (Tables 5, 6) (Fig. 2a). We observed that our COVID-19 patients with low FT3 serum values showed an increased Na_*e*_:K_*e*_ ratio, compared to COVID-19 patients with normal FT3 serum values (Table 6). In addition, an inverse correlation of FT3 serum values and Na_*e*_:K_*e*_ ratio was found in all COVID-19 and non-COVID-19 thyroid outpatients (Fig. 3b), similar to that observed with hydration. These results indicated that COVID-19 patients with low FT3 serum values had both marked water and salt retention in a picture resembling that observed in severe hypothyroid patients with myxedema.

**Table 5:** Bioelectrical impedance analysis results for non-COVID-19 outpatients with thyroid diseases

	Rz (W)	Xc (W)	TBW (%)	ECW (%)	ICW (%)	Na_*e*_:K_*e*_ (ratio)	PhA (°)
Non-COVID-19 outpatients with thyroid diseases (n. 96)							
Subclinical hyperthyroidism (n. 19)	561.5 ± 96.0	44.7 ± 9.8	52.2 ± 6.7	53.8 ± 7.7	46.2 ± 7.7	1.3 ± 0.3	4.7 ± 1.1
Euthyroidism (n. 57)	540.3 ± 78.3	49.0 ± 17.1	51.2 ± 6.7	50.9 ± 6.1	49.1 ± 6.1	1.1 ± 0.2	5.1 ± 1.2
Subclinical hypothyroidism (n. 12)	552.3 ± 104.6	49.6 ± 16.0	55.0 ± 7.0	50.9 ± 4.7	49.1 ± 4.7	1.2 ± 0.3	5.1 ± 0.8
Overt hypothyroidism (n. 7)	522.6 ± 87.8	47.7 ± 10.4	52.2 ± 8.3	49.9 ± 7.0	50.7 ± 7.0	1.2 ± 0.3	5.3 ± 1.3
Myxedema (n. 1)	459	29	57.2	60.2	39.8	1.57	3.6

**Table 6:** Bioelectrical impedance analysis results for COVID-19 patients in ICU

COVID-19 patients (n. 74)	Rz (W)	Xc (W)	TBW (%)	ECW (%)	ICW (%)	Na_*e*_:K_*e*_ (ratio)	PhA (°)
Normal FT3 serum levels > 1.7 pg/ml (n. 32)	453.6 ± 123.8	31.6 ± 14.5	55.7 ± 10.7	59.2 ± 13.1	40.9 ± 13.1	1.8 ± 1.2	4.1 ± 1.9
Low FT3 serum levels ± 1.7 pg/ml (n. 42)	382.8 ± 113.5	21.7 ± 14.8^*^	59.5 ± 7.4^*^	65.2 ± 14.2^*^	34.8 ± 14.2^*^	2.4 ± 1.5^*^	3.2 ± 2.0

Rz: resistance; Xc: reactance; TBW: total body water; ECW: extracellular water; ICW: intracellular water; Na_*e*_:K_*e*_: sodium/potassium exchangeable ratio; PhA: 50 kHz Whole Body Phase Angle

*p < 0.05

### Lower FT3 serum values in COVID-19 patients are associated with serum hypernatremia

To further analyzed the correlation between sodium and potassium and thyroid function in our COVID-19 patients, we measured the serum levels of thyroid hormones and of Na^+^ and K^+^ in the same day we performed BIA analysis. We found that serum Na^+^ levels were slightly but significantly elevated in COVID-19 patients with low FT3 serum values, compared to those with normal FT3 serum values (Table 7). We couldn’t detect any significant correlation between Na_*e*_:K_*e*_ ratio, measured by BIA, and the serum levels of both Na^+^ and K^+^.

**Table 7:** Na_*e*_:K_*e*_ ratio compared to Na^+^ and K^+^ in the serum of COVID-19 patients in ICU

	BIA	Serum	
	Na_*e*_:K_*e*_ ratio	Na^+^ (mmol/l)	K^+^ (mmol/l)
COVID-19 patients (n. 74)			
Normal FT3 serum levels > 1.7 pg/ml (n. 32)	1.8 ± 1.2	139.4 ± 3.7	4.0 ± 0.4
Low FT3 serum levels ± 1.7 pg/ml (n. 42)	2.4 ± 1.5*	141.2 ± 6.0*	4.1 ± 0.6

Na_*e*_:K_*e*_: sodium/potassium exchangeable ratio

*p < 0.05

### Statistical analysis

The two study groups, namely COVID-19 patients with low and normal FT3 serum values, didn’t differ in terms of age, gender or BMI. On the other side, the non-COVID-19 patients, are significantly different. COVID-19 patients are older and composed of more men, compared to the group of outpatients with thyroid diseases (Tables 1 and 2). However, the group of patients with thyroid diseases at different functional conditions was chosen to examine the effects of thyroid functional status on BIA parameters and to test the specificity of the results observed in the context of COVID-19 with NTIS. To study the potential correlations among the considered variables in the sample, we performed matrix analysis of all pairwise correlations on the whole sample (data not shown). We found that ECW, PhA, Na_*e*_:K_*e*_ ratio and hydration are highly correlated one each other. We, therefore, focused our attention to two of them and namely the hydration and the Na_*e*_:K_*e*_ ratio.

Regarding the multivariable analysis, the following tables report the point estimates, 95% confidence intervals (95% CI) and *p*-values of a multivariable linear regression estimated on all patients. Tables 8 and 9 summarize the main significant clinical variables associated with FT3 for each group when hydration (Table 8) and Na_*e*_:K_*e*_ (Table 9) were included in the model. In both cases, TSH, hydration and Na_*e*_:K_*e*_ were negatively associated with FT3. It is also clear that being infected by COVID-19 seems to significantly reduce FT3 levels.

**Table 8:** Multivariable linear regression model in the all sample

Predictors	Estimates	95% CI	*P*-value
(Intercept)	4.10	2.95 to 5.24	< 0.001*
TSH	− 0.07	− 0.13 to − 0.01	0.024*
TBW/FFM (hydration)	− 0.02	− 0.03 to − 0.00	0.040*
COVID-19	− 1.10	− 1.34 to − 0.87	< 0.001*

95% CI: 95% confidence interval

*p < 0.05

**Table 9:** Multivariable linear regression model in the all sample

Predictors	Estimates	95% CI	*p*-value
(Intercept)	3.04	2.87 to 3.20	< 0.001*
TSH	− 0.06	− 0.12 to − 0.00	0.036*
Na_*e*_:K_*e*_	− 0.11	− 0.21 to − 0.01	0.026*
COVID-19	− 1.16	− 1.36 to − 0.95	< 0.001*

95% CI: 95% confidence interval

*p < 0.05

Finally, we estimated univariable and multivariable logistic regression models fitted on COVID-19 Positive patients to evaluate the factors associated with low levels of FT3 (FT3 ≤ 1.7 pg/ml) and normal levels of FT3, (FT3 > 1.7 pg/ml). At univariable analysis, only TSH (0.011 p-value), Na_*e*_:K_*e*_ (0.070 p-value) and hydration (0.024 p-value) were significantly positively and negatively associated with FT3 serum levels, respectively. At multivariable analysis, the stepwise selection procedure begins with a full model containing all candidate predictor variables. Then, according to the AIC values, gender, ECW and age are discarded from the model in the first three step of the procedure, followed by PhA, BMI, hydration and FT4. At the end of the selection process, the final model includes TSH and Na_*e*_:K_*e*_. By looking at the estimated Odds Ratios, 95% CIs and *p*-values in Table 10, one can see that for a unit increase in TSH we expect a 91% increase in the odds of having normal levels of FT3. Meanwhile, the effect of increasing Na_*e*_:K_*e*_ involves a reduction of 40% in the odds of having normal levels of FT3.

**Table 10:** Multivariable logistic regression model fitted on COVID-19 Positive patients

Predictors	Odds ratios	95% CI	*p*-value
(Intercept)	1.11	0.40–3.12	0.838
TSH	1.91	1.16–3.16	0.014*
Na_*e*_:K_*e*_	0.60	0.38–0.96	0.035*

95% CI: 95% confidence interval

*p < 0.05

All together, these data indicate that there is a significant negative association between FT3 serum levels and increase in the Na_*e*_:K_*e*_ ratio and hydration. These results suggest that T3 action at the periphery may play a crucial role in the development of ionic imbalance and of the anasarcatic condition, associated with the progression of the COVID-19 in patients hospitalized in ICUs.

### The mRNA expression of the ATP1A1 gene is reduced in the PBMC of COVID-19 patients with NTIS

**Fig. 4:**
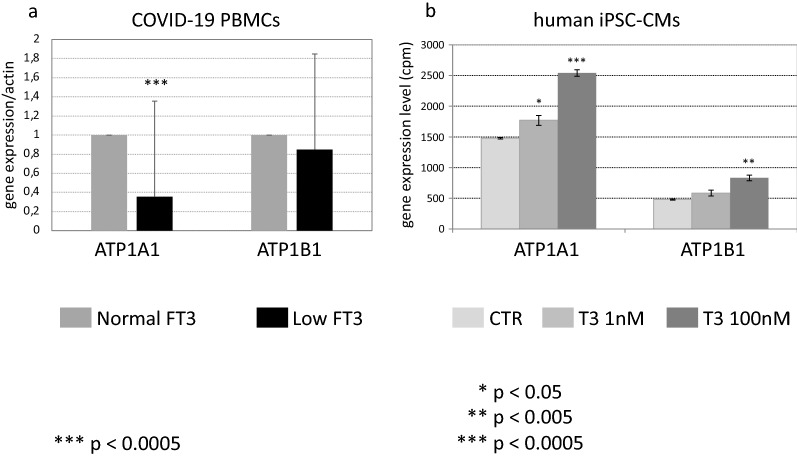
mRNA expression levels of the genes ATP1A1 and ATP1B1 in the PBMC of COVID-19 (**a**) presenting normal FT3 (gray bars) and low FT3 serum values (black bars) and in hiPSC-CMs (**b**), treated with T3 at 1 nM (gray bars) and 100 nM (dark gray bars). Results are compared to those obtained in untreated hiPSC-CMs (white bars), used as controls (CTR) (light gray bars). Data have been retrieved from GEO public functional genomics data repository (GEO accession code GSE172348). *p < 0.05, **p < 0.005, ***p < 0.0005

We measured the expression levels of the two genes coding for the two major isoforms of the Na^+^/K^+^ pump in the PBMC obtained from 47 COVID-19 patients during the acute phase of the disease. The results of our RT-PCR analysis are reported (Fig. 4a). COVID-19 patients with NTIS showed reduced expression levels of both genes, compared to COVID-19 patients with normal FT3 serum values. In particular, the mean reduction in the expression of the ATP1A1 gene in PBMCs of COVID-19 with NTIS compared to that observed in COVID-19 patients without NTIS was more evident (64.5% reduction) and statistically significant (*p* < 0.0005) with respect to that observed in the expression of the ATP1B1 gene (15.2% reduction). These results indicate that low FT3 serum values are associated with downregulation of the expression of this pump, with the ATP1A1 emerging as the mostly deregulated gene.

### The mRNA expression of the ATP1A1 and of ATP1B1 genes are increased in hiPSC-CMs upon T3 treatment

To confirm the ability of T3 to modulate the expression of these genes, we retrieved the results concerning the expression of the two genes ATP1A1 and ATP1B1 from the RNA-seq analysis performed in human induced pluripotent stem cell-derived cardiomyocytes (hiPSC-CMs) (GEO accession code GSE172348). These cells were treated with T3 at two different final concentrations, 1 and at 100 nM respectively. Both genes showed a progressive increase in their expressions upon T3 treatment (Fig. 4b), indicating that T3 is able to regulate the expression of these genes in the human cardiac cell model, with the ATP1A1 gene, encoding for the ATPase Na^+^/K^+^ transporting subunit α 1, emerging as the mostly responsive to T3 treatment. Altogether data from this experiment and those obtained in the PBMC of COVID-19 patients reinforce the hypothesis that T3 could represent a master regulator of this pump, potentially involved in the development of the anasarcatic condition observed in our COVID-19 patients with NTIS.

## Discussion

During the actual COVID-19 pandemic there has been concern among the endocrinologists, and in particular among the thyroidologists, regarding the possibility that the pandemic situation could affect the clinical management of thyroid patients [22]. Many International Endocrine and Thyroid Resources and Societies released statements and furnished guidance on how to treat and manage thyroid patients during the COVID-19 pandemic. They include the American Thyroid Association [23], the European Thyroid Association [24], the British Thyroid Foundation [25], the European Society of Endocrinology [26], and the American Association for Clinical Endocrinologists [27]. However, there is another way to look at the situation. The question, in fact, could be different and, precisely, we may ask: is there any influence of thyroid function on the clinical course of COVID-19? The clinical outcome of critically ill patients may be compromised by many different conditions, including reduced blood volume because of gastrointestinal bleeding or the use of diuretics, reduced ventilatory drive because of intercurrent pulmonary infections, sepsis, impairment of the central nervous system regulatory mechanisms due to stroke, congestive heart failure due to myocardial infarction and the use of sedatives, or hyponatremia. Among all these causes, the occurrence of reduced serum levels of FT3 has been reported to be associated with unfavorable clinical course and eventually death [28]. Demonstration of low FT3 serum levels is a frequent event in patients hospitalized in ICUs and it has been reported to be present in up to 70–80% of them [29, 30]. It has been reported also in critically ill children [31].

From the clinical point of view, there is strikingly similarity between the clinical condition encountered in critically ill patients with NTIS and the condition known for a long time as myxedema. Myxedema was described for the first time by Sir William Withey Gull in 1874 [32] and named by William M. Ord soon later [33]. It is characterized by abnormal deposition of mucopolysaccharides in the cutis and dermis, which results in swelling of the affected area. In patients with longstanding severe untreated hypothyroidism myxedema may be associated with marked alteration of the water metabolism with fluid retention, and development of an anasarcatic status. Many years ago, by using a dilution technique and a radioactive tracer, it has been reported that patients affected by myxedema had increased total body water and Na_*e*_ [2]. These patients often show pleural, peritoneal and pericardial effusion and may progress toward a deep state of prolonged unconsciousness till occurrence of coma [34]. The key features of myxedema coma are an altered mental status, consisting in somnolence and lethargy that may develop via stupor into a comatose state, and precipitating event including cold exposure, infections, drugs such as diuretics, tranquillizers, sedatives, analgesics, trauma, stroke, heart failure, gastrointestinal bleeding [35]. Usually, myxedematous coma develops during winter months and the typical patient is an older woman with altered consciousness, and a history of hypothyroidism, neck surgery or radioactive iodine treatment. In critically ill patients, homeostasis is disrupted by precipitating events too and may develop a life-threatening clinical condition resembling the one observed in patients with myxedema coma [36]. The major difference between myxedema and NTIS relies in the rapidity of evolution. In classical myxedema, due to longstanding and untreated hypothyroidism, the evolution of the symptoms is slowly progressive [37], while in critically ill patients, hospitalized in ICU, the evolution of a NTIS is usually fast and may rapidly lead to unfavorable lethal outcome.

Since the occurrence of low FT3 concentrations in healthy subjects during fasting was considered an adaptive response to protect the organism against deleterious catabolic consequences of lack of macronutrients [38, 39], much attention was given to evaluate the nutritional status of patients with NTIS [40]. Few studies have focused their attention to the hydration status too.

Low FT3 serum values were reported to be independently associated with 28-day mortality in a prospective observational study focused on a large cohort of patients (n. 305) admitted to the ICU [41]. The occurrence of NTIS in COVID-19 patients has been reported in retrospective studies performed in small cohort of patients [42, 43]. The impact of NTIS on disease severity was also assessed in children with COVID-19 [44]. In our University Hospital we have started an extensive study on epidemiological, associated comorbidities, clinical, laboratory and molecular features of patients with COVID-19 [45–48]. In addition, we reviewed the EU negotiations with pharmaceutical companies and EU strategy for mass COVID-19 vaccination campaign [49].

We demonstrated that reduced levels of FT3 serum levels can be considered as a severity disease biomarker of COVID-19 [11]. Our results are in agreement with those reported by another group [12]. The correlation between FT3 serum levels and COVID-19 has prompted some researchers to initiate a new phase II randomized, double blind, placebo controlled trial (Thy-Support,ClinicalTrials.gov, Identifier: NCT04348513) for enhancing recovery of critically ill COVID-19 patients treated with T3 [50]. However, the primary endpoint of this clinical trial is to determine whether the administration of intravenous T3 facilitates weaning from cardiorespiratory support, compared to placebo. The results of our present study indicate that the analysis of the hydroelectrolytic balance at the periphery, measured by BIA, could represent a better indicator in the evaluation of the potential benefit of treatment with T3.

The first description of BIVA was published by Piccoli et al. [51] and it is now largely applied in the assessment of two compartment body composition, for nutritional purposes in sport, geriatric medicine and in clinical medicine [52]. Several BIA indexes and parameters are considered useful to assess the nutritional status in critically ill patients too where they represent significant prognostic factors [53]. Although multifrequency bioelectrical impedance (MF-BIA) was reported to have a high level of reliability [54], comparison of both methods showed agreement without significant proportional errors [55]. The vectorial bioimpedance analysis (BiaVector) is a validated system, able to interpret the measured single components of impedance vector Z at 50 kHz, and to represent them in a probabilistic graph of vector BIA. BIA has the advantage of being noninvasive, portable, inexpensive, practical, fast, safe and easy to use and it is widely applied in clinical practice to the assessment of body composition, in terms of evaluation of the nutrition and hydration state [56]. Although it is considered a valid tool to assess body fluid composition and distribution in subclinical hypothyroidism, when compared to dilution methods [57], there are not many studies in the literature concerning the relationship of body composition measured by BIA in thyroid diseases [58]. Most of them are focused on hyperthyroidism [59], on short term hypothyroidism [60] or on subclinical hypothyroidism [57]. To the best of our knowledge, BIA has never been applied to severe hypothyroidism, myxedema and to NTIS. In the literature there is only one study, performed in dialytic patient in Spain, in which an attempt was made to correlate the serum FT3 levels to different parameters of malnutrition and inflammation, including anthropometric measurements and BIA [61]. However, the medium FT3 values in these patients was normal (4.0 ± 0.71 pmol/l, ranging from 3.95 to 6.80 pmol/l). In this study, the authors reported that patients with low FT3 in serum showed a reduced muscular and cellular mass, with no difference in their fat mass. A correlation was found between arm circumference and FT3 serum levels. However, no mention was made regarding the hydration status of these patients.

Several reports indicate the utility of BIA in the evaluation of critically ill patients in ICUs [62, 63] and our study on the application of BIA in ICU for the assessment of critically ill patients is in agreement with such studies. However, in addition to this we report also the significant correlation among BIA parameters and the occurrence of NTIS.

Among the different parameters evaluated by BIA, the Phase Angle (PhA) represents an indicator of cell membrane damage and body cell mass. It is a useful, independent indicator for predicting the malnutrition risk in hospitalized patients affected by different pathologies and is considered a suitable tool for the assessment of nutritional status of hospitalized patients with different co-morbidities. It has been used as a nutritional indicator in different patient groups, including hemodialysis patients [64–66], hospitalized children [67] as well as in geriatric patients [68]. In addition, it has been used in the evaluation of the nutritional state of patients affected by different types of cancer [69–73]. Finally, PhA was associated with mortality in elderly patients [74]. In particular, it has been reported that reduced PhA values may predict a decrease in survival in many different diseases [75]. Our COVID-19 patients showed reduced levels of PhA and those with very low FT3 serum values showed a more intense reduction in the PhA values. BIA allows also the assessment of fluid distribution in the body. Water represents the largest chemical component in mammals and is an essential component of living organisms. Water plays a central role in nutrient transport, waste removal, maintenance of cell volume, and thermal regulation. In adult mammals the fraction of fat-free body mass (FFM) as water is the best-known body-composition constant. It was initially analyzed in guinea pigs [76] and then found to be remarkably stable at 0.73 in several mammal species, even if they show remarkable difference in their body sizes [77]. It is widely used to estimate total body fat in vivo and it is measured as a fraction of TBW and FFM [21]. TBW can be measured by dilution techniques using isotopes [78] and by BIA.

It is well known that one of the most important consequence of thyroid hormone deficiency consists in the accumulation of hydrophilic molecules, hyaluronic acid and other glycosaminoglycans in interstitial tissue, responsible for the occurrence of interstitial edema in the skin and swelling of the affected area. However, the accumulation of water is not restricted to the skin and can be observed also in striated and heart muscle [79]. Since edema represents a common manifestation of hypothyroidism, the evaluation of FFM hydration, measured as the fraction of FFM as water, namely the TBW/FFM ratio, could represent an ideal peripheral indicator of thyroid hormone function. In this regard, it has been reported that bioelectrical resistance was found to be a better indicator of thyroid function than anthropometry in healthy subjects [80]. However, in a small cohort of patients (16 patients with subclinical hypothyroidism and 15 with normal thyroid function), subclinical hypothyroidism had no effect on compartments of body fluids [57]. Our results obtained in the control subjects with thyroid diseases at different functional conditions confirm this observation. We couldn’t detect any significant difference in the hydration of patients with subclinical or overt hypothyroidism. We found an increase in TBW/FFM ratio as well as in Na_*e*_:K_*e*_ ratio only in the single patient with severe hypothyroidism and myxedema, showing low FT3 serum levels. The same results observed in our single myxedematous patient were seen in critically ills patients showing NTIS. We found a significant inverse correlation between FT3 values and TBW/FFM ratio in these patients (Fig. 3a). Another relevant parameter is represented by the Na_*e*_:K_*e*_ exchangeable ratio and it was inversely correlated with FT3 values too (Fig. 3b). Total exchangeable sodium (Na_*e*_), total exchangeable potassium (K_*e*_) and TBW are the major determinants of the plasma water sodium concentration [81]. Recently, they have been integrated with some additional physiologic determinants, including the osmotically inactive exchangeable Na^+^ and K^+^ and the osmotically active non-Na^+^ and non-K^+^ osmoles [82]. The Na_*e*_:K_*e*_ ratio, measured by BIA, has been proposed as an objective marker of nutritional status and an excellent indicator of an adverse clinical outcome. It has been used to identify malnourished patients at risk of dying [83]. The abnormal sodium storage has been related to the severity of COVID-19 and sodium removal by drugs as well as reduction in sodium intake has been suggested to improve outcomes of COVID-19 patients [84]. In this regards, experimental data indicate that intervention to improve hydration reduces mortality due to acute respiratory distress syndrome (ARDS) [85]. Moreover, it has been suggested that TBW balance i.e., body water retention, may represent a crucial risk factor for COVID-19 mortality [86]. Since our results indicate that one of the possible targets of T3 action could be represented by the Na^+^/K^+^ pump, we analyzed the expression levels of the two major isoforms of the α and β subunits of the ATPase Na^+^/K^+^ transporting gene that codes for the Na^+^/K^+^ pump. The Na^+^/K^+^ is an ion pump whose function is to create and maintain an electrochemical gradient across the plasma membrane and is critical for the resting membrane potential, electrical activity of muscle and nerve, Na^+^-coupled transport, transepithelial transport, nutrient uptake, osmotic balance and cell volume regulation [87, 88]. Its activity ensures the exit of sodium ions out of the cell in exchange for potassium ions entering the cell. Thyroid hormones are known to be able to modulate the activity of the ATPase Na^+^/K^+^ transporting gene in the peripheral tissues [89]. In particular, it has been reported that they are able to modulate its expression at the transcriptional level [90]. Experimental studies, performed in cultured chick cardiac myocytes, indicated that inhibition of Na^+^/K^+^ pump promotes the efflux of ions and water, causing cell shrinkage [91]. Our results are in agreement with such studies and indicate that the expression of the two major subunits of the ATP1 gene, namely the ATP1A1 and the ATP1B1, are downregulated in the PBMCs of our COVID-19 patients with low FT3 serum values, as compared to those with normal FT3 serum values. In particular, the ATP1A1 emerges as the most downregulated gene in patients with low FT3 serum levels. In addition, we demonstrated that, in hiPSC-CMs, T3 treatment is able to enhance, in a dose-dependent manner, the expression of these two genes and, in particular, of the ATP1A1 gene. These results indicate that the Na^+^/K^+^ pump is regulated, at the transcriptional level, by T3, and reinforce the hypothesis that its downregulation may be involved in the development of the anasarcatic condition associated with the NTIS in our COVID-19 patients. It is interesting to note that many studies have indicated that conditions as hypertension, diabetes, coronary artery disease, chronic heart failure, chronic kidney disease, obesity, and advanced age significantly worsen the prognosis in COVID-19. Indeed, all these clinical entities share a common denominator that is represented by the abnormal sodium retention. Moreover, the excessive sodium accumulation may lead to tissue inflammation [92]. Clinical conditions associated with sodium-retaining states are responsible for a more severe and sometimes fatal clinical course of COVID-19 [84]. Recently, the role of sodium retention is gaining attention both to explain the clinical severity in COVID-19 patients and as a therapeutic target to improve the outcome of COVID-19 patients [84, 93]. However, although there are some reports postulating a possible role in driving alveolar epithelial barrier failure in severe COVID-19 [94] and some years ago a crucial role of the ATP1A1 α subunit in coronavirus infection was reported, suggesting potential therapeutic implications [95], few studies have focused the attention on the expression/activity of the Na/K pump so far. This is somehow surprising considering that this pump is responsible for a basic relevant function, namely the maintenance of the physiological electrolyte gradient and regulation of the influx/efflux of both sodium and potassium across the cellular membranes. In addition, this pump plays a relevant role in relaying cardiotonic steroid-binding-induced signals into cells [96] and, most importantly, in edema clearance [97]. Most attention have been given to the upstream renin–angiotensin–aldosterone system (RAAS). Our study indicates that besides the RAAS, another hormonal mechanism could be involved in the abnormal sodium retention, and this mechanism could be under the control of the T3. The reductions of FT3 serum levels were specifically associated to hydroelectrolytic balance alterations in COVID-19 patients with NTIS as compared to the control group of COVID-19 patients without NTIS. In addition, they were not detected in our external group of non-COVID-19 outpatients, affected by several different thyroid diseases, including hypothyroid patients. This suggests that such alterations occur only in the context of NTIS associated to COVID-19 and, presumably, to other critical illness. A similar pattern of hydroelectrolytic alterations was observed only in one patient that presented myxedema, due to severe and prolonged hypothyroidism. We postulate that the reduced levels of serum FT3, observed in our COVID-19 patients with NTIS, are responsible for the increase in the Na_*e*_:K_*e*_ ratio measured by BIA, as well as in the increased of the serum sodium levels. The use of BIA in COVID-19 patients allowed us to demonstrate that the occurrence of NTIS in these patients has severe consequences on hydroelectrolytic balance, a picture that shows strikingly similarities to that observed in myxedema, although in COVID-19 patients the process evolves more rapidly. Based on these results, NTIS should be considered as an acute and severe hypothyroid condition at the periphery that would require appropriate treatment.

## Conclusions

In conclusion, we demonstrated that the occurrence of NTIS in COVID-19 patients hospitalized in intensive care units is responsible for severe alteration of the hydroelectrolytic balance that can be easily measured by BIA. These alterations were specifically observed in critical ill COVID-19 patients with NTIS and were not seen in non-COVID-19 outpatients affected by thyroid diseases at different functional conditions, except for the single patient with myxedema. In COVID-19 patients, reduced FT3 serum levels were significantly associated with increased TBW and ECW as well as reduced ICW. In addition, Na*e*:K*e* ratio was increased in COVID-19 with NTIS, indicating that T3 is a major regulator of these changes and it might exert its action through one of its longtime known target, namely the Na^+^/K^+^ ATPase gene. NTIS observed in our patients is a condition that appears similar to that caused by severe hypothyroidism and known as myxedema. Based on the results of our study, we recommend the use of BIA in severe/critical COVID-19 patients to monitor hydroelectrolytic balance at the periphery. Our study indicates that hydration and Na_*e*_:K_*e*_ ratio, measured by BIA, could represent optimal endpoints in the evaluation of the efficacy of T3 treatment in an ongoing interventional clinical trial.

## Data Availability

The datasets used and/or analyzed during the current study are available from the corresponding author on reasonable request. All data generated or analyzed during this study are included in this published article and its Additional files.

## Abbreviations

COVID-19: Coronavirus disease 2019
ARDS: Acute respiratory distress syndrome
NTIS: Nonthyroidal Illness Syndrome
ICU: Intensive care unit
BIA: Bioelectrical Impedance Analysis
SF-BIA: Single-Frequency Bioelectrical Impedance Analysis
Rz: Resistance
Xc: Reactance
PhA: Phase angle
Z: Impedance
BCM: Body cell mass
TBW: Total body water
ECW: Extra cellular water
ICW: Intra cellular water
Na_*e*_:K_*e*_: Na:K exchange rate
FFM: Fat-free mass
FM: Fat mass
SOFA: Sequential Organ Failure Assessment score
PBMC: Peripheral blood mononuclear cells
hiPSC-CMs: Human induced pluripotent stem cell-derived cardiomyocytes
rRT-PCR: Real-time reverse-transcription polymerase chain reaction
ATP1A1: ATPase Na^+^/K^+^ transporting subunit α 1
ATP1B1: ATPase Na^+^/K^+^ transporting subunit β 1
RAAS: Renin–angiotensin–aldosterone system
